# The reporting of neuropsychiatric symptoms in electronic health records of individuals with Alzheimer’s disease: a natural language processing study

**DOI:** 10.1186/s13195-023-01240-7

**Published:** 2023-05-12

**Authors:** Willem S. Eikelboom, Ellen H. Singleton, Esther van den Berg, Casper de Boer, Michiel Coesmans, Jeannette A. Goudzwaard, Everard G. B. Vijverberg, Michel Pan, Cornalijn Gouw, Merel O. Mol, Freek Gillissen, Jay L. P. Fieldhouse, Yolande A. L. Pijnenburg, Wiesje M. van der Flier, John C. van Swieten, Rik Ossenkoppele, Jan A. Kors, Janne M. Papma

**Affiliations:** 1 Department of Neurology and Alzheimer Center Erasmus MC, Erasmus MC University Medical Center, PO Box 2040, 3000 CA Rotterdam, the Netherlands; 2 Department of Neurology, Alzheimer Center Amsterdam, Amsterdam University Medical Centers, Amsterdam, the Netherlands; 3 Department of Psychiatry, Erasmus MC University Medical Center, Rotterdam, the Netherlands; 4 Department of Internal Medicine, Section of Geriatrics, Erasmus MC University Medical Center, Rotterdam, the Netherlands; 5 Clinical Memory Research Unit, Lund University, Malmö, Sweden; 6 Department of Medical Informatics, Erasmus MC University Medical Center, Rotterdam, the Netherlands

**Keywords:** Alzheimer’s disease, Apathy, Affective symptoms, Diagnosis, Machine learning, Neuropsychiatric symptoms, Prevalence

## Abstract

**Background:**

Neuropsychiatric symptoms (NPS) are prevalent in the early clinical stages of Alzheimer’s disease (AD) according to proxy-based instruments. Little is known about which NPS clinicians report and whether their judgment aligns with proxy-based instruments. We used natural language processing (NLP) to classify NPS in electronic health records (EHRs) to estimate the reporting of NPS in symptomatic AD at the memory clinic according to clinicians. Next, we compared NPS as reported in EHRs and NPS reported by caregivers on the Neuropsychiatric Inventory (NPI).

**Methods:**

Two academic memory clinic cohorts were used: the Amsterdam UMC (*n* = 3001) and the Erasmus MC (*n* = 646). Patients included in these cohorts had MCI, AD dementia, or mixed AD/VaD dementia. Ten trained clinicians annotated 13 types of NPS in a randomly selected training set of *n* = 500 EHRs from the Amsterdam UMC cohort and in a test set of *n* = 250 EHRs from the Erasmus MC cohort. For each NPS, a generalized linear classifier was trained and internally and externally validated. Prevalence estimates of NPS were adjusted for the imperfect sensitivity and specificity of each classifier. Intra-individual comparison of the NPS classified in EHRs and NPS reported on the NPI were conducted in a subsample (59%).

**Results:**

Internal validation performance of the classifiers was excellent (AUC range: 0.81–0.91), but external validation performance decreased (AUC range: 0.51–0.93). NPS were prevalent in EHRs from the Amsterdam UMC, especially apathy (adjusted prevalence = 69.4%), anxiety (adjusted prevalence = 53.7%), aberrant motor behavior (adjusted prevalence = 47.5%), irritability (adjusted prevalence = 42.6%), and depression (adjusted prevalence = 38.5%). The ranking of NPS was similar for EHRs from the Erasmus MC, although not all classifiers obtained valid prevalence estimates due to low specificity. In both cohorts, there was minimal agreement between NPS classified in the EHRs and NPS reported on the NPI (all kappa coefficients < 0.28), with substantially more reports of NPS in EHRs than on NPI assessments.

**Conclusions:**

NLP classifiers performed well in detecting a wide range of NPS in EHRs of patients with symptomatic AD visiting the memory clinic and showed that clinicians frequently reported NPS in these EHRs. Clinicians generally reported more NPS in EHRs than caregivers reported on the NPI.

**Supplementary Information:**

The online version contains supplementary material available at 10.1186/s13195-023-01240-7.

## Introduction

Over 80% of the individuals who visit the memory clinic in the early clinical stages of Alzheimer’s disease (AD) experience neuropsychiatric symptoms (NPS) such as apathy, depressive symptoms, irritability, and sleep disturbances [1–3]. These symptoms are associated with poor clinical outcomes including reduced quality of life [4], increased caregiver burden [5], and a faster disease progression [6].

Clinicians working at the memory clinic strongly rely on proxy-based instruments such as the Neuropsychiatric Inventory (NPI) to diagnose NPS in AD [7–9]. However, proxy-based NPS instruments are subject to recall bias and can be affected by the mood, fatigue, knowledge, and cultural beliefs of informal caregivers who usually provide the information [10, 11]. Therefore, the perspective of clinicians on NPS may provide a valuable addition to the impression of caregivers [11, 12]. However, little is known about how clinicians perceive and report NPS in the memory clinic setting. Electronic health records (EHRs) may provide a unique opportunity to address this question. Clinicians working at the memory clinic document symptoms, observations, outcomes of the diagnostic work-up, and differential diagnoses as free-text descriptions in EHRs. This unstructured format allows to report on complex clinical phenomena while taking the nuances of the individual patient into account [13] and are increasingly used for research purposes to study clinical care practices, the manifestation of complex clinical symptoms, and the natural disease course [14, 15].

The advantage that free-text descriptions in EHRs offer simultaneously conveys a major challenge to structurally and systematically examine unstructured free text [16]. As the manual assessment of EHRs is very time-consuming, natural langue processing (NLP) applications are increasingly used to automatically assign particular categories to phrases in free text. These applications only require a selection of EHRs to be manually rated by experts, i.e., annotated [13, 16]. Based on these annotations, NLP algorithms are trained and validated in order to automatically classify the remaining EHRs [16].

Recently, NLP applications have been used to detect NPS in EHRs of older adults with cognitive impairment [17–19]. These studies have shown that NLP applications can identify older adults at increased risk for dementia based on NPS presence [18], estimate NPS prevalence based on EHRs in individuals with dementia [17, 19], and indicate potential underdiagnosis of NPS in dementia [17]. So far, NLP applications have not been used in the memory clinic setting and previous studies have only focused on agitation, affective symptoms, and psychotic symptoms [17–19], while neglecting other NPS that are also common in the early clinical stages of AD such as apathy, irritability, and sleeping behavior [1, 2]. Furthermore, memory clinics primarily establish NPS in AD by the impression of clinicians and/or using proxy-based instruments such as the NPI [7]. Yet, no prior study investigating the use of NLP to detect NPS has incorporated comparisons of results to NPI outcomes.

The aim of this study was to use NLP to estimate the reporting of a wide range of NPS reported by clinicians in EHRs of individuals with mild cognitive impairment (MCI) or AD dementia at the memory clinic.

## Methods

This study was approved by the Medical Ethics Committees of the Erasmus MC (2018–1137) and the Amsterdam UMC (2021.0044).

### Data

All EHRs were obtained from 3001 individuals who visited the Alzheimer Center Amsterdam at the Amsterdam University Medical Centers between March 1993 and December 2020 [20] and from 646 patients who visited the Alzheimer Center Erasmus MC at the Erasmus MC University Medical Center between January 2004 and April 2019. Patients were selected if they had a clinical diagnosis of MCI [21], AD dementia [22], or mixed AD/vascular dementia (VaD) [22]. All individuals with MCI visiting the Alzheimer Center Amsterdam were amyloid-beta positive based on either cerebrospinal fluid analysis [23] or visual rating of an amyloid-beta PET scan [24], while individuals with MCI visiting the Alzheimer Center Erasmus MC were only selected if they had AD as suspected primary etiology based on clinical impression, neuroimaging, and/or cerebrospinal fluid profile. In both samples, a subsample of the individuals with a clinical diagnosis of AD dementia had cerebrospinal fluid or amyloid-beta PET scan available indicating amyloid-beta positivity (65% in Alzheimer Center Amsterdam, 32% in the Alzheimer Center Erasmus MC).

EHRs from both hospitals contained free-text information on the referral, medical history, clinical impression, neurological examination, physical assessment, medication review, and psychiatric evaluation. There were also EHRs written by neuropsychologists describing history taking, clinical impression, and neuropsychological test performances. EHRs from the Alzheimer Center Amsterdam were written by neurologists or neuropsychologists, while EHRs from the Alzheimer Center Erasmus MC were written by neurologists, geriatricians, or neuropsychologists. For each patient, the EHRs from these different clinicians created within a three-month period were clustered as this was the time usually needed to establish a clinical diagnosis. A random selection of 500 EHRs from the Alzheimer Center Amsterdam was used for the training set and internal validation, while a random sample of 250 EHRs from the Alzheimer Center Erasmus MC was used for external validation.

The NPI or its questionnaire form (NPI-Q) assessed as part of the diagnostic work-up were used [25, 26]. For the intra-individual comparison, we denoted an NPI or NPI-Q domain score ≥ 1 as the presence of a specific NPS.

### Data annotation

Ten trained clinicians independently annotated the data. The raters consisted of four psychologists, two neurologists (in training), two psychiatrists (in training), one clinical neuropsychologist, and one geriatrician. The training set of 500 EHRs was divided into five sets of 100 EHRs that were independently annotated by two raters. Four of these raters also annotated the test set of 250 EHRs, divided into two sets of 125 EHRs each annotated by two raters. The pairs were selected such that they differed in terms of background and years of clinical experience.

In an iterative process, two raters (W.S.E., M.P.) developed a guideline for the annotation of 13 NPS categories of which 12 categories were analogous to the 12 NPI domains [25]. We added a 13th category for general terms that describe nonspecific NPS including but not limited to “behavioral and psychological symptoms of dementia,” “changes in behavior,” and “challenging behavior.” Each of these categories was described in detail in the annotation guideline that was based on existing assessment scales, criteria for neuropsychiatric syndromes in dementia, and clinical experience. All ten raters tested the annotation guideline in 20 EHRs from the Alzheimer Center Amsterdam and 10 EHRs from the Alzheimer Center Erasmus MC that were not part of the training and test set. Hereafter, a consensus meeting was held with all raters discussing any disagreements. The final annotation guideline was established based on this discussion (see Additional file 1 for a translated version).

Annotations were made with the web-based annotation tool *brat* [27]. Raters were instructed to mark the word, phrase, or sentence that described an NPS and to label it with one of the 13 categories. After annotating the EHRs independently, each rater pair discussed the annotations where they initially disagreed and decided on a final consensus annotation. If needed, a third rater was consulted to reach consensus.

### Text preprocessing

Different preprocessing steps were tested including stop word removal (using the Dutch stop word list in the R package *stopwords*), stemming (reducing words to their canonical form using the Dutch stemmer in R package *SnowballC*), and removal of phrases that indicated negations (e.g., “no depressive symptoms”). After preprocessing, the remaining free-text was divided into unigrams and bigrams, i.e., sequences of one or two words, which were used as features to train each classifier [28].

### Classifier training

We used NLP to assign categories to free text [13], i.e., the classification of 13 NPS categories in EHRs. The annotations by the raters were used to train a classifier for each NPS category. We developed a binary classifier to determine the presence or absence of that category in an EHR. Generalized linear classifiers (method glmnet in the R package *caret*) were trained and internally validated on the training set using tenfold cross-validation. The performance of the classifiers was externally validated on the test set.

### Statistical analysis

#### Evaluation of annotations and classifier performance

Different inter-annotator agreement scores were derived from the annotations for each NPS category across all five pairs of raters, including accuracy (proportion of agreement) and the kappa coefficient (*κ*, proportion of agreement corrected for chance agreement).

The performance of each classifier was evaluated by comparing its automated classification of NPS with the manual annotations by the raters with the area under the receiver operating characteristic curve (AUC) on the training set using tenfold cross-validation and on the external test set. An AUC 0.70–0.80 was considered acceptable, an AUC 0.80–0.90 was considered excellent, and an AUC > 0.90 was considered outstanding [29]. For each classifier, sensitivity and specificity were calculated and a probability cutoff was selected by maximizing the Youden index.

#### Prevalence of NPS in EHRs

Only classifiers that had good diagnostic abilities (AUC ≥ 0.80) were included in subsequent analyses. The prevalence of each NPS category in the EHRs across patients was estimated for both cohorts separately using the classifiers. We estimated the prevalence and calculated confidence intervals taking the sensitivity and specificity of each classifier into account to correct for imperfect classifiers [30].

#### Intra-individual comparison between EHRs and NPI

Intra-individual comparisons of the NPS classified in EHRs and NPS reported on the NPI were conducted in a subsample of individuals who had an NPI assessment available. For each NPS, we assessed the agreement between NPS reported in EHRs and NPS according to the NPI using the kappa coefficient. Of all the patients who had a particular NPS reported in their EHR, we calculated the proportion of patients with that NPS not endorsed on the NPI (EHR + NPI-). Similarly, of all the patients who had a particular NPS endorsed on the NPI, we calculated the proportion of patients with that NPS not reported in their EHR (EHR-NPI +).

## Results

### Patient characteristics

The majority of the patients included in both cohorts were diagnosed with AD dementia (78.4%), approximately half were female (52%), and the majority was White (90%) (Table 1). The patients from the Alzheimer Center Amsterdam were younger, a smaller proportion had MCI, and a higher proportion had an AD-biomarker confirmed diagnosis compared with the patients from the Alzheimer Center Erasmus MC (all *p* < 0.001, Table 1).

**Table 1:** Characteristics of the memory clinic cohorts

	**Alzheimer Center Amsterdam**	**Alzheimer Center Erasmus MC**
***N* patients**	3001	646
**Age, mean (SD)**^**a**^	67.2 (8.6)	71.1 (9.3)***
**Sex, *N* (%) female**	1571 (52.4%)	323 (50.0%)
**Education, median (IQR)**^b^	5.0 (2.0)	5.0 (2.0)
**Whites, *N* (%)**^**c**^	189 (90.0%)	345 (89.1%)
**MMSE, mean (SD)**^**d**^	21.1 (6.4)	21.6 (5.5)
**Diagnosis, *N* (%)**		
**Mild cognitive impairment**	436 (14.5%)	157 (24.3%)***
**AD dementia**	2438 (81.2%)	422 (65.3%)***
**AD/VaD dementia**	127 (4.2%)	67 (10.4%)***
**Amyloid-beta positive, *N* (%)**^**e**^	2092 (69.7%)	184 (28.5%)***
**NPI or NPI-Q available, *N* (%)**	2022 (67.4%)	133 (20.6%)***

*Abbreviations*: *AD* Alzheimer’s disease, *MMSE* Mini-Mental State Examination, *NPI* Neuropsychiatric Inventory, *NPI-Q* Neuropsychiatric Inventory questionnaire, *VaD* vascular dementia

^***^ p < 0.001

^a^Data missing for *n* = 79 (Erasmus MC)

^b^Dutch education system categorized into (1) less than 6 years primary education [< 6 years], (2) completed primary education [6 years], (3) more than 6 years of primary education, without a secondary school diploma [8 years], (4) lower vocational training [9 years], (5) advanced vocational training or lower professional education [10–11 years], (6) advanced professional training or upper secondary school [12–18 years], and (7) academic degree [> 18 years]. Data missing for *n* = 261 (Amsterdam UMC) and *n* = 306 (Erasmus MC)

^c^Data missing for *n* = 2792 (Amsterdam UMC) and *n* = 259 (Erasmus MC)

^d^Data missing for *n* = 253 (Amsterdam UMC) and *n* = 125 (Erasmus MC)

^e^Based on either cerebrospinal fluid (i.e., amyloid-beta_42_ < 550 pg/mL or tau/amyloid-beta_42_ ratio > 0.52) or visual rating of an amyloid-beta PET scan

### Annotations

For the training set, the median accuracy of the five pairs of raters across all NPS was 0.94 (range 0.92–0.96), and the median kappa coefficient across all NPS suggested moderate agreement (*κ* = 0.71, range *κ* = 0.49–0.74). There was low agreement between raters for aberrant motor behavior (median *κ* = 0.35), euphoria (median *κ* = 0.49), disinhibition (median *κ* = 0.52), and agitation (median *κ* = 0.54), while agreement was obtained for hallucinations (median *κ* = 0.99) and general descriptions of NPS (median *κ* = 0.94) (Additional file 2; Supplemental Table 1). For the external test set, the overall accuracy scores (0.94, 0.91) and the overall kappa coefficients (*κ* = 0.71, *κ* = 0.74) for the two pairs of raters were highly comparable to the training set (Additional file 2; Supplemental 1). It was not possible to train a classifier for euphoria as this NPS was annotated in only five EHRs in the training set (1.0% of EHRs in training set).

### Performance of classifiers

The cross-validated performance of the classifiers was excellent, with AUCs ranging from 0.81 to 0.91 (Table 2). The sensitivity and specificity of all classifiers were > 0.70, except for the specificity of the classifier for aberrant motor behavior (0.61).

**Table 2:** Performance of the classifiers for the training and test set

	**Training set**				**Test set**			
NPS category	**AUC**	**Sensitivity**	**Specificity**	**Youden’s Index**	**AUC**	**Sensitivity**	**Specificity**	**Youden’s Index**
Eating behavior	0.91	0.85	0.81	0.66	0.83	0.59	0.94	0.52
Anxiety	0.90	0.78	0.89	0.66	0.84	0.60	0.92	0.52
Depression	0.90	0.71	0.97	0.68	0.88	0.79	0.85	0.64
Disinhibition	0.90	0.91	0.77	0.68	0.80	0.67	0.84	0.51
Irritability	0.89	0.83	0.83	0.66	0.84	0.76	0.84	0.61
Apathy	0.88	0.88	0.80	0.68	0.84	0.91	0.61	0.52
Delusions	0.88	0.79	0.87	0.67	0.75	0.69	0.78	0.47
Sleeping behavior	0.88	0.88	0.78	0.66	0.83	0.76	0.78	0.54
Agitation	0.87	0.87	0.83	0.70	0.81	0.63	0.90	0.53
Hallucinations	0.87	0.78	0.96	0.74	0.67	0.40	0.96	0.36
NPS general	0.87	0.80	0.92	0.72	0.93	0.85	0.90	0.75
AMB	0.81	0.91	0.61	0.52	0.51	0.61	0.51	0.12

*Abbreviations*: *AMB* aberrant motor behavior, *AUC* area under the curve, *NPS* neuropsychiatric symptom

For the external test set, classifiers performance yielded AUCs ranging from 0.51 to 0.93. Although AUC values decreased compared to the training set (median AUC difference − 0.06, range − 0.30 to + 0.06), most AUCs remained excellent (AUC > 0.80), except for delusions (AUC = 0.75), hallucinations (AUC = 0.67), and aberrant motor behavior (AUC = 0.51). Therefore, these three NPS were not included in subsequent analyses. The sensitivity of most classifiers was substantially lower for the external test set, with a sensitivity > 0.70 for only the classifiers of apathy, general descriptions of NPS, depressive symptoms, irritability, and sleeping behavior. The specificity of most classifiers was similar or higher in the external test set compared to the training set, except for aberrant motor behavior (training set 0.61 vs. test set 0.51) and apathy (0.80 vs. 0.61) (Table 2).

### Prevalence of NPS in EHRs

**Fig. 1:**
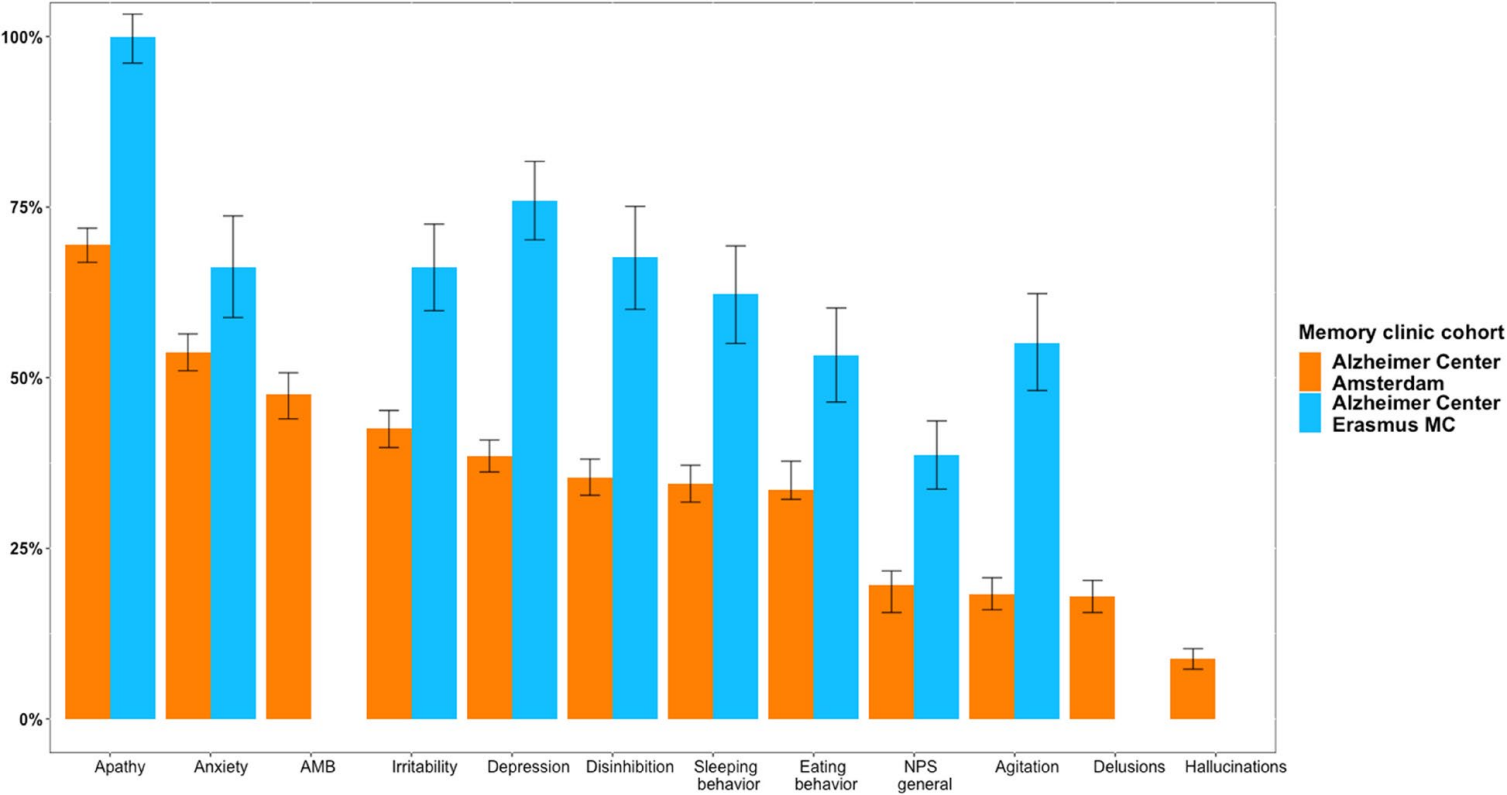
Adjusted prevalence of neuropsychiatric symptoms classified in electronic health records. *Abbreviations*: *AMB*, aberrant motor behavior; *NPS*, neuropsychiatric symptoms. Prevalence estimates were adjusted for bias due to imperfect test [30]. All adjusted prevalence rates were significantly higher in the Alzheimer Center Erasmus MC compared to Alzheimer Center Amsterdam (all FDR-adjusted *p* < 0.001). Classifiers for aberrant motor behavior, delusions, and hallucinations were not used in Alzheimer Center Erasmus MC data as AUC < 0.80

The most prevalent NPS classified in the EHRs of patients who visited the Alzheimer Center Amsterdam were apathy (adjusted prevalence = 69.4%) and anxiety (adjusted prevalence = 53.7%), followed by aberrant motor behavior (adjusted prevalence = 47.5%), irritability (adjusted prevalence = 42.6%), and depressive symptoms (adjusted prevalence = 38.5%) (Fig. 1). The majority of the prevalence estimates was lower when adjusted for the sensitivity and specificity of the classifiers but did not change substantially (mean difference: − 4.7 percentage point, range − 16.2 to + 9.3%) (Additional file 2; Supplemental Table 2).

All adjusted prevalence rates of NPS in EHRs of patients visiting the Alzheimer Center Erasmus MC were significantly higher compared to Alzheimer Center Amsterdam (all FDR-adjusted *p* < 0.001) (Fig. 1). Still, the ranking of most common NPS in EHRs of the Alzheimer Center Erasmus MC was similar to the Alzheimer Center Amsterdam: apathy (adjusted prevalence = 100.0%), depressive symptoms (adjusted prevalence = 75.9%), anxiety (adjusted prevalence = 66.2%), and irritability (adjusted prevalence = 66.2%). Adjusting for the sensitivity and specificity of the classifiers when applied in the external test set substantially changed the prevalence estimates (mean difference: + 12.3 percentage point range − 0.3 to + 23.8%; Additional file 2; Supplemental Table 2).

The prevalence of NPS classified in EHRs differed significantly according to sex and disease severity (Additional file 2; Supplemental Tables 4 and 5). EHRs of male patients contained more general descriptions of NPS than EHRs of female patients in both cohorts (FDR-adjusted *p* < 0.05). At the Alzheimer Center Amsterdam, agitation, aberrant motor behavior, apathy, disinhibition, irritability, and sleeping behavior were more often reported in males (all FDR-adjusted *p* < 0.001), while EHRs of females contained more reports of anxiety and depression (all FDR-adjusted *p* < 0.01). We found similar findings for agitation, anxiety, depression, and disinhibition in the Alzheimer Center Erasmus MC dataset (all FDR-adjusted *p* < 0.001) (Additional file 2; Supplemental Table 4). In addition, EHRs of patients with MCI contained more reports of anxiety and depression compared to EHRs of patients with dementia (all FDR-adjusted *p* < 0.01), while delusions and hallucinations were more common in patients with dementia compared to patients with MCI at the Alzheimer Center Amsterdam (all FDR-adjusted *p* < 0.001). At the Alzheimer Center Erasmus MC, depression and disinhibition were more often reported in EHRs of patients with MCI than in EHRs of patients with dementia (all FDR-adjusted *p* < 0.01) (Additional file 2; Supplemental Table 5).

To evaluate the accuracy of the adjusted classifier estimates, estimates were compared with annotations for the training set and the external test set (Additional file 2; Supplemental Table 3). Generally, NPS prevalence rates based on adjusted classifiers were highly comparable to the annotations. However, several adjusted prevalence rates in the Alzheimer Center Erasmus MC data set were not valid probably due to low specificity (e.g., 100.0% [96.1–103.3%] for apathy).

### Intra-individual comparison between EHRs and NPI assessments

**Fig. 2:**
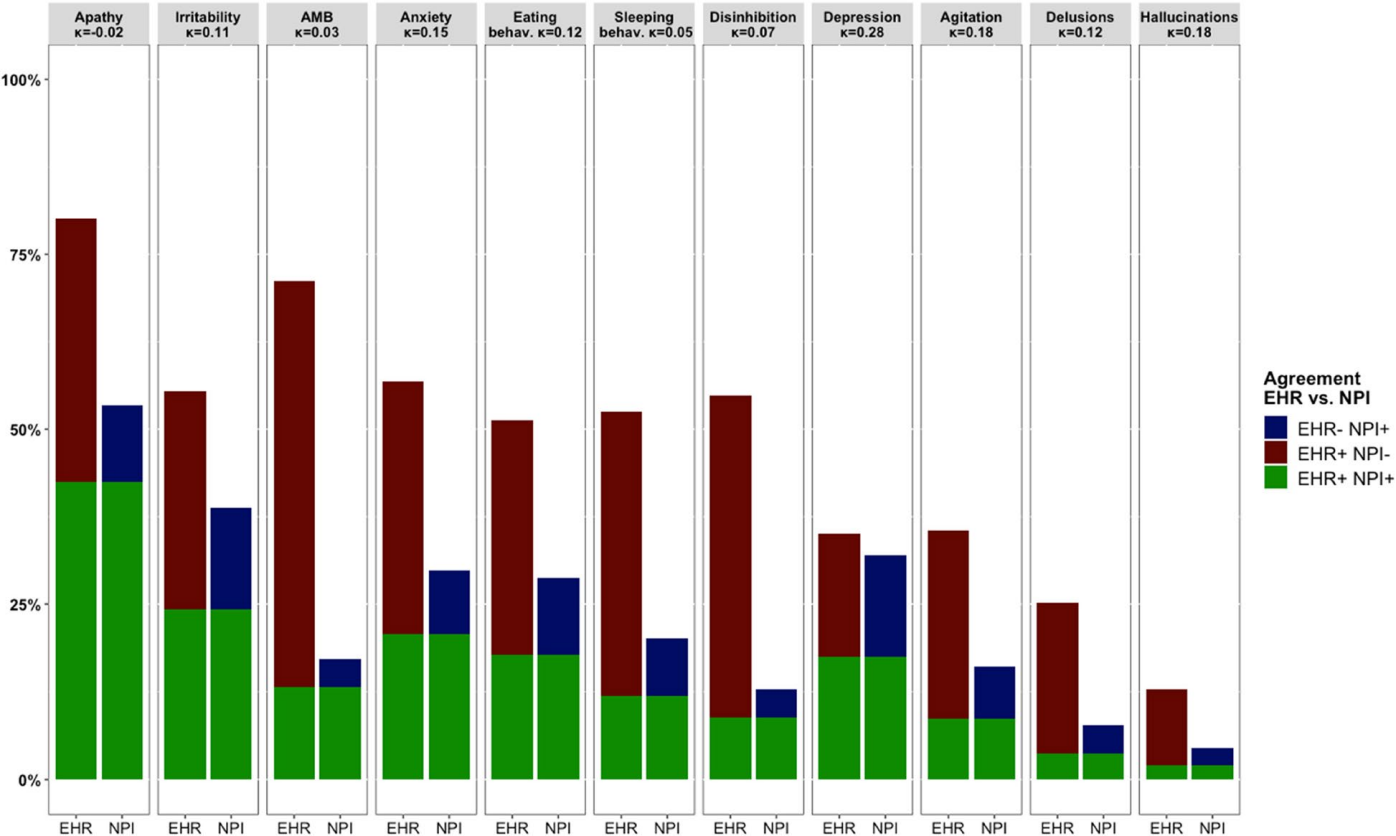
Agreement between NPS described in EHRs and reported on NPI in Alzheimer Center Amsterdam cohort (*n* = 2022). Abbreviations: *AMB*, aberrant motor behavior; *EHR*, electronic health record; *EHR-NPI +*, NPS reported on NPI; but not described in EHR; *EHR + NPI-*, NPS described in EHR; but not reported on NPI; *EHR + NPI +*, NPS reported on both EHR and NPI; *Κ*, Kappa coefficient; *NPI*, Neuropsychiatric Inventory; *NPS*, neuropsychiatric symptoms

**Fig. 3:**
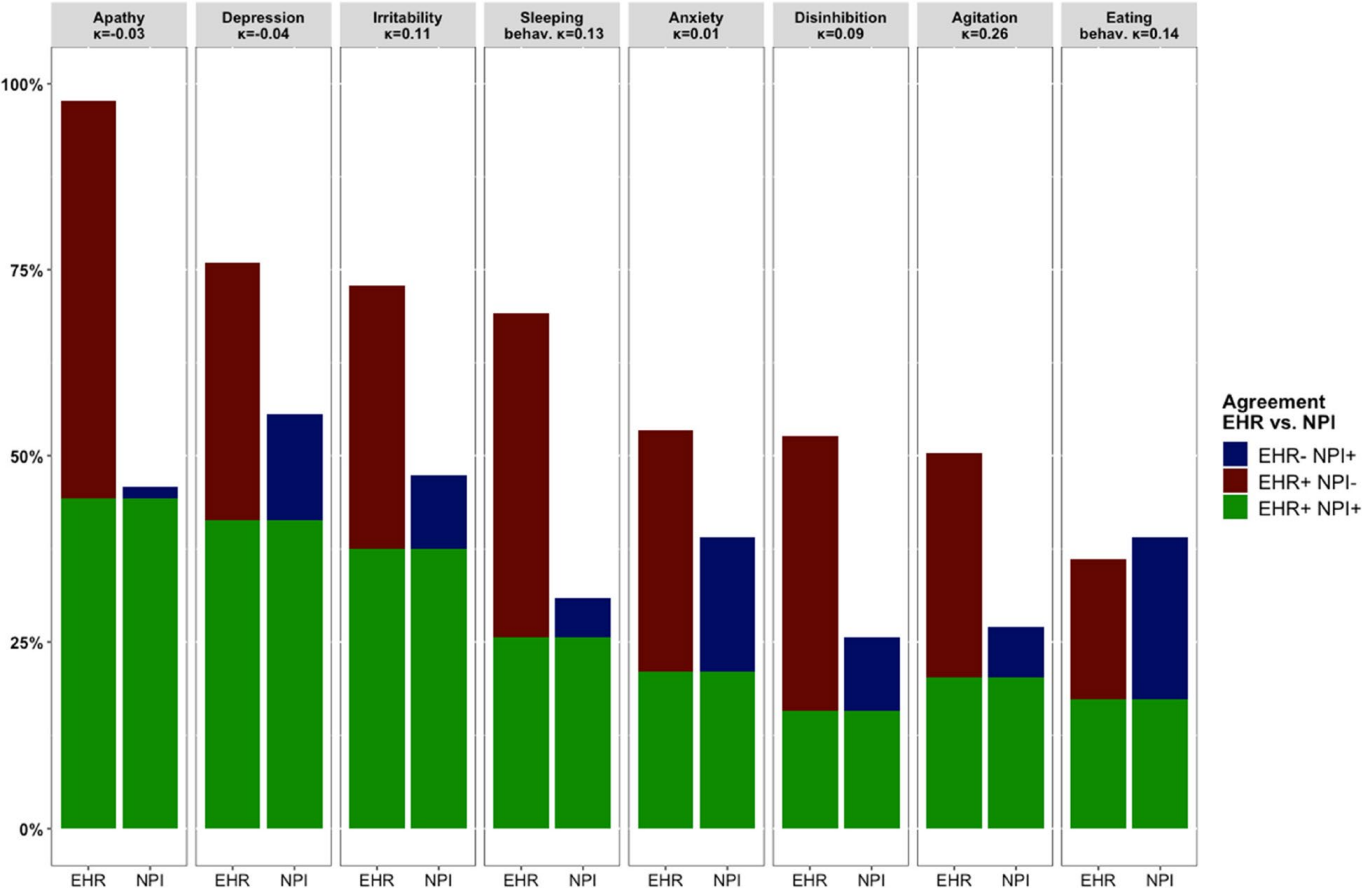
Agreement between NPS described in EHRs and reported on NPI in Alzheimer Center Erasmus MC cohort (*n* = 133). *Abbreviations*: *AMB*, aberrant motor behavior, *EHR*, electronic health record, *EHR-NPI +*, NPS reported on NPI, but not described in EHR, *EHR + NPI-*, NPS described in EHR, but not reported on NPI, *EHR + NPI +*, NPS reported on both EHR and NPI, *Κ*, Kappa coefficient, *NPI*, Neuropsychiatric Inventory, *NPS*, neuropsychiatric symptoms. Classifiers for aberrant motor behavior, delusions, and hallucinations were not included in these analyses as AUC < 0.80

A subsample of 2022 individuals (67%) from the Alzheimer Center Amsterdam and 133 individuals (21%) from the Alzheimer Center Erasmus MC had an NPI assessment available. For both cohorts, the overall prevalence of NPS in EHRs was considerably higher than NPS reported on the NPI (Alzheimer Center Amsterdam median prevalence 52.5% vs. 20.1%; Alzheimer Center Erasmus MC 62.8% vs. 39.1%) (Figs. 2 and 3).

Kappa coefficients indicated minimal to no agreement between NPS described in the EHRs by clinicians and NPS reported on the NPI by caregivers (Figs. 2 and 3). Agreement was minimal for depressive symptoms in the Alzheimer Center Amsterdam (*κ* = 0.28) and agitation (*κ* = 0.26) in the Alzheimer Center Erasmus MC, while there was no agreement between all other NPS reported by clinicians and caregivers (all *κ* < 0.18). Kappa coefficients were highly similar across the two cohorts, except for a lower agreement for depressive symptoms (*κ* =  − 0.04) and anxiety (*κ* = 0.01) in the Alzheimer Center Erasmus MC compared to the Alzheimer Center Amsterdam (depression *κ* = 0.28, anxiety *κ* = 0.15).

Figures 2 and 3 show that the disagreements between NPS described in the EHRs by clinicians and NPS reported on the NPI by caregivers were mostly due to an lower NPS prevalence rates according to the NPI (i.e., EHR + NPI-), as approximately 30% of the patients had a symptom solely reported in their EHR. Yet, NPS were solely reported on the NPI for almost 10% the patients (i.e., EHR-NPI +).

## Discussion

Main findings of this study were that (1) NLP classifiers performed well in detecting a wide range of NPS in EHRs of patients with symptomatic AD visiting the memory clinic, although the generalizability of some NLP classifiers to detect NPS in EHRs in an external data set was limited; (2) clinicians frequently described NPS in EHRs of patients with symptomatic AD in both memory clinic cohorts; and (3) there was low agreement between NPS in EHRs reported by clinicians and NPS on NPI assessments reported by caregivers.

### Performance of classifiers

Based on the AUCs (range 0.81–0.91), performance of the classifiers was considered excellent in the training set and comparable to previous NLP studies in dementia [31]. External validation of classifiers showed good generalizability for the majority of NPS, except for hallucinations, delusions, and aberrant motor behavior. The few previous studies that used NLP to detect NPS have not conducted external validation [17–19], similar to the studies that used machine learning approaches recently reviewed in the field of geriatric psychiatry [31]. Hence, performing such analyses was considered a clear strength of this study as external validation is essential to establish the generalizability of classifiers [13].

### Prevalence of NPS in EHRs

Adjusting for imperfect sensitivity and specificity generally yielded accurate NPS prevalence rates when compared to annotated NPS. However, this resulted in extreme high values for some classifiers in the external data set (e.g., 100.0% [96.1–103.3%] for apathy), questioning the use of these classifiers in an external data set. A possible explanation is the moderate inter-rater agreement scores, probably due to substantial variation in terminologies used to denote NPS among clinicians [32–35]. Several researchers have raised concerns that divergent terminologies may hamper adequate recognition and treatment of NPS [32, 35], while it remains unknown to which degree this affects all NPS observed in AD. Our findings suggest that the clinicians’ abilities to uniformly detect NPS was especially limited for aberrant motor behavior, euphoria, disinhibition, and agitation, while higher agreement was observed among clinicians for NPS such as hallucinations, delusions, and depressive symptoms. The implementation of the use of diagnostic criteria for NPS such as agitation may help to uniform the nomenclature used by clinicians working at the memory clinic [36].

The adjusted prevalence estimates indicated that clinicians frequently reported NPS in EHRs of individuals with symptomatic AD visiting the memory clinic, especially apathy, anxiety, irritability, aberrant motor behavior, and depressive symptoms. These symptoms are commonly diagnosed in the early clinical stages of AD based on proxy-based measures, self-report instruments, and clinician rating scales [2, 3, 37]. The adjusted prevalence estimates of hallucinations, delusions, depressive symptoms, and agitation in our study were lower compared to prevalence rates in EHRs reported in two previous NLP studies [17, 19]. These two studies clustered symptoms that were analyzed separately in our study (e.g., delusions and hallucinations). Furthermore, these studies also included EHRs of individuals with severe dementia living in nursing homes, which may explain the higher NPS prevalence rates reported. In addition, in contrast to previous studies [17, 19], our study adjusted for imperfect classification performances of the classifiers which generally reduced prevalence estimates.

We found a similar ranking of NPS reported in EHRs in both memory clinic cohorts included. Yet, we observed substantial higher prevalence estimates across all NPS in EHRs of patients who visited the Erasmus MC that might result from several factors. First, this might be due to the limited classification abilities of the classifiers for this external data set with a tendency to overestimate NPS, e.g., 100.0% (95% CI 96.1–103.3%) for apathy. Second, data collection for the Alzheimer Center Amsterdam started in 1993, while we have data from the year 2004 onwards from the Alzheimer Center Erasmus MC. Awareness that NPS are a core clinical feature of NPS has increased among clinicians in later years [38, 39], which might have resulted in higher NPS prevalence rates in the Alzheimer Center Erasmus MC. When selecting EHRs from the Alzheimer Center Amsterdam written between 2004 and 2020, we found a significant increase in prevalence estimates of all NPS (all FDR-adjusted *p* < 0.05), except for hallucinations. However, prevalence estimates of all NPS remained significantly higher for EHRs of the Alzheimer Center Erasmus MC (all FDR-adjusted *p* < 0.001), while only the prevalence of anxiety was similar for both centers (FDR-adjusted *p* > 0.05, Additional file 2; Supplemental Table 6). Therefore, these differences may arise from systematic differences in patient populations, also reflected by significant differences in NPI assessments between centers (Additional file 2; Supplemental Table 7). The Alzheimer Center Erasmus MC is a frontotemporal dementia (FTD) center of expertise. Consequently, a large proportion of the patients referred to this center are suspected of having FTD due to substantial NPS including agitation, disinhibition, and psychotic symptoms.

The prevalence of NPS as reported by clinicians in EHRs was related to the sex of the patient in both cohorts. The increased prevalence of depressive symptoms among females and apathy among males are in line with the findings of a recent meta-analysis on sex differences in NPS in AD dementia as primarily assessed by proxy-instruments such as the NPI [40]. In addition, NPS prevalence in EHRs was also associated with disease severity. Psychotic symptoms were more common in EHRs of patients with dementia compared to MCI, which is in line with prior research [2, 37]. In contrast, affective symptoms were more common in EHRs of patients with MCI, which has also been reported previously [3].

### Comparison between EHRs and NPI assessments

We found at best minimal agreement between NPS that were described in EHRs by caregivers and NPS endorsed on the NPI by caregivers. It is important to note that NPS were spontaneously described or observed and reported in EHRs by clinicians, while NPS were assessed using a structured and standardized assessment tool in caregivers. Given these differences in NPS reports, we cannot directly compare the perspectives of clinicians and caregivers regarding their NPS impression, though both of these methods are used to indicate the presence of specific NPS in the memory clinic.

Our findings do corroborate with prior studies showing large disagreement between clinicians and caregivers in standardized NPS instrument outcomes [12, 41–43]. Discrepancies in NPS ratings might result from differences in the reference point based on which clinicians and caregivers consider certain behaviors abnormal. For instance, caregivers have to indicate whether behaviors are abnormal compared to pre-morbid functioning, while clinicians usually evaluate behaviors while referring to the general population and/or their personal clinical experience. In addition, prior research suggests substantial differences in nomenclature used to describe NPS between caregivers and clinicians [32].

Clinicians generally reported more NPS in EHRs than caregivers reported on the NPI. Clinicians may be less biased by factors that are known to affect proxy-based NPS instruments such as mood, stress, fatigue, and recall bias [10]. In addition, NPS that were described in EHRs were not limited to specific wording and a timeframe of four weeks that is usually assessed with the NPI [25]. Finally, it should be noted that NPS were detected in EHRs based on imperfect classifiers with a tendency to overestimate the NPS prevalence. Although caregivers generally reported less NPS, a notable proportion of NPS that caregivers endorsed on the NPI were not mentioned in EHRs. A recent study by our group suggests that NPS may be underrecognized by memory clinic physicians as they experience difficulties diagnosing NPS that mainly occur at home and because some physicians do not perceive NPS as core feature of the early clinical stages of AD [44].

No gold standard exists to establish the presence of NPS in AD. Therefore, we cannot make firm conclusions about the comparison between NPS reports by caregivers and clinicians. It is imperative to relate NPS ratings of clinicians and caregivers to alternative and possibly less subjective measures of NPS, e.g., using wearables such as actigraphy [45]. However, wearable may only be able to capture abnormalities in motor activity as seen in apathy, agitation, aberrant motor behavior, and sleeping behavior. These applications might not be suitable to assess NPS such as depression, delusions, and hallucinations that consist of changes in feelings, thoughts, and perception.

### Implications of findings

Our findings have important implications. First, although no gold standard exists, our findings may suggest that caregivers and clinicians report different NPS in community-dwelling individuals with symptomatic AD. This has serious consequences as memory clinic clinicians strongly rely on proxy-based instruments to establish the presence of NPS and to evaluate the effectiveness of pharmacological and non-pharmacological interventions [7]. Moreover, proxy-based instruments are commonly used as outcome measure in clinical trials targeting NPS in AD [8]. Future studies should pair proxy-based NPS instruments with clinician-based instrument such as the NPI-C [11]. Second, the developed classifiers might be used to study the manifestation of NPS in EHRs of populations without cognitive deficits as a growing body of research suggests that NPS may precede cognitive impairment during the course of AD [1, 46]. Third, although the performance of a proportion of the classifiers was not considered sufficient to classify individual patients in the external test set at this stage, improving classification abilities holds promise for clinical practice. For example, these NLP applications might be used to identify patients in the early clinical stages of AD with significant NPS in other care settings than memory clinics, e.g., primary care. Hereby, these patients may be referred to a specialized memory clinic to receive adequate treatment as primary care providers have reported substantial difficulties in detecting and treating NPS [47, 48]. As NPS manifest differently in pre-dementia populations [49], prevalence estimates of the developed classifiers should be compared with manual annotations in a subset when applied in pre-dementia populations.

### Strengths and limitations

Strengths of this study include (1) the large well-defined cohort of individuals with symptomatic AD, of which a large proportion had a clinical diagnosis supported by AD-biomarkers; (2) a large team of trained clinicians who independently annotated a wide range of NPS using a guideline; and (3) the external validation of the classifiers using an external memory clinic cohort. This study also has certain limitations that should be considered. First, the two cohorts studied were academic memory clinic populations with an overrepresentation of White and highly educated patients and young-onset and atypical variants of AD dementia. As considerable differences were already noted between these two cohorts in terms of NPS prevalence rates, future studies are needed to study the prevalence of NPS in EHRs of people in the early clinical stages of AD visiting memory clinics of general hospitals and other care settings. In addition, the limited performance of several classifiers might be explained by the low number of samples that were used to train the classifiers [50]. Moreover, our study indicated a lack of consistent nomenclature for NPS among clinicians which hampered the annotation process. Future studies may explore the use of word embeddings, such as generated with *word2vec* in the annotation process to identify different but semantically similar terms and also as features to enhance the classifiers [51]. Finally, we were not able to take the severity and clinical relevance of NPS reported in EHRs into account. Instead, the mere presence of NPS in EHRs was annotated and used in all analyses. To align this with NPI assessments, we compared NPS reported in EHRs with NPI domain scores ≥ 1. However, this may have led to the inclusion of changes in behavior and emotions that may be trivial and of little clinical significance. Therefore, future studies are needed that take the severity of NPS reported by clinicians in EHRs into account, e.g., by examining the number of NPS reported in one EHR and/or by training separate classifiers for each NPS according to symptom severity. Note that findings did not change when comparing NPS classified in EHRs with NPI scores domain scores ≥ 4 indicating clinically relevant NPS (Additional file 2; Supplemental Table 8).

## Conclusions

Clinicians frequently report NPS in EHRs of individuals with symptomatic AD visiting the memory clinic. Within patients, we found low agreement between NPS reported in EHRs by clinicians and NPS reported on the NPI by caregivers, with substantially more NPS reported by clinicians than caregivers. More research is needed to determine whether this implies that caregivers underestimate NPS or clinicians overestimate NPS.

## Supplementary Information

**Additional file 1.** Translated annotation guide.

**Additional file 2.** Additional analyses. **Supplemental Table 1.** Number of final annotations, accuracy, and kappa coefficients for the training set and the external test set. **Supplemental Table 2.** Unadjusted and adjusted prevalence rates of NPS classified in EHRs. **Supplemental Table 3.** NPS prevalence across EHR based on annotations and classifiers. **Supplemental Table 4.** NPS classified in EHRs according to sex of the patient. **Supplemental Table 5.** NPS classified in EHRs according to disease severity. **Supplemental Table 6.** NPS classified in EHR according to year of visit. **Supplemental Table 7.** Comparison of NPI assessments between centers. **Supplemental Table 8.** Kappa coefficients for NPS classified in EHRs vs. NPS reported on NPI according to NPI cut off.  

## Data Availability

The datasets used and/or analyzed during the current study are available from the corresponding author on reasonable request.
