# Supplementary material for: The reporting of neuropsychiatric symptoms in electronic health records of individuals with Alzheimer’s disease: a natural language processing study
Source: Alzheimers Res Ther. 2023 May 12;15:94. doi: 10.1186/s13195-023-01240-7 (PMC10176879; doi:10.1186/s13195-023-01240-7)
Supplement: Supplementary file 1 — Additional file 1. Translated annotation guide. [file 13195_2023_1240_MOESM1_ESM.pdf]

Annotation Guide to annotate neuropsychiatric symptoms in Electronic Health Records of patients with Alzheimer's disease

Willem S. Eikelboom,[a] Ellen H. Singleton,[b] Esther van den Berg,[a] Casper de Boer,[b] Michiel Coesmans,[c] Jeannette A. Goudzwaard,[d] Everard G.B. Vijverberg,[b] Michel Pan,[a] Cornalijn Gouw,[c] Merel O. Mol,[a] Freek Gillissen,[b] Jay L.P. Fieldhouse,[b] Yolande A.L. Pijnenburg,[b] Wiesje M. van der Flier,[b] John C. van Swieten,[a] Rik Ossenkoppele,[b,e] Jan A. Kors,[f] Janne M. Papma,[a]

[a] *Department of Neurology and Alzheimer Center Erasmus MC, Erasmus MC University Medical Center, Rotterdam, the Netherlands*
[b] *Department of Neurology, Alzheimer Center Amsterdam, Amsterdam University Medical Centers, Amsterdam, the Netherlands*
[c] *Department of Psychiatry, Erasmus MC University Medical Center, Rotterdam, the Netherlands*
[d] *Department of Internal Medicine, Section of Geriatrics, Erasmus MC University Medical Center, Rotterdam, the Netherlands*
[e] *Clinical Memory Research Unit, Lund University, Malmö, Sweden*
[f] *Department of Medical Informatics, Erasmus MC University Medical Center, Rotterdam, the Netherlands*

Corresponding author
Willem S. Eikelboom, PhD
Department of Neurology, Erasmus MC University Medical Center
PO Box 2040, 3000 CA, Rotterdam, the Netherlands
w.eikelboom@erasmusmc.nl

This annotation guide was based on:
- Neuropsychiatric Inventory (NPI)
- Behavioral pathology in Alzheimer's disease (BEHAVE-AD)
- Revised Memory and Behavior Checklist (RMBC)
- Brief Psychiatric Rating Scale (BPRS)
- Cohen-Mansfield agitation inventory (CMAI)
- Geriatric depression scale (GDS)
- Cornell scale for depression in dementia (CSDD)
- Apathy Evaluation Scale (AES)
- Rating anxiety in dementia (RAID)
- Diagnostic criteria for apathy in neurocognitive disorders (Miller et al., 2021, Alzheimers Dement)
- Agitation in cognitive disorders: Progress in the International Psychogeriatric Association consensus clinical and research definition (Sano et al., 2023, Int Psychogeriatrics)
- Revisiting criteria for psychosis in Alzheimer's disease and related dementias: toward better phenotypic classification and biomarker research (Fischer et al., 2020, J Alzheimers Dis)
- Provisional diagnostic criteria for depression of Alzheimer disease: rationale and background (Olin et al., 2002, Am J Geriatr Psychiatr)
- Diagnostisc and Statistical Manual of Mental Disorders, fifth edition (DSM-5)
- Dutch guidelines for diagnosis and treatment of neuropsychiatric symptoms in dementia (e.g. *Probleemgedrag bij mensen met dementie*)
- 20 EHRs from the Alzheimer Center Amsterdam and 10 EHRs from the Alzheimer Center Erasmus MC that were not part of the training and test set
- Consensus among authors

The original annotation guide was developed to annotate Dutch text and was translated into English for publication in Alzheimer's Research & Therapy. Please note that NPS terminologies may differ for the Dutch and English language, and that subtleties of the Dutch language may have been lost in the translation.

1 Delusions

Definition

Delusions are beliefs or assumptions that are false and cannot be corrected.

Terminologies and examples

1. Suspicion
He/she is suspicious
Suspicious that caregiver is leaving patient
Does not trust partner/child/informal carer
Convinced/thinks/feeling/idea that partner, child and/or other caregiver is unfaithful or a cheater
Convinced/thinking/feeling/the idea of being cheated
Angry at partner/child/caregiver because of infidelity
The idea that others/strangers steal/hide stuff
Believing/thinking/feeling/the idea that the place one lives in is not his/her home

2. Conspiracy allegations
Believing/thinking/feeling/the idea of being haunted, poisoned, infected
Convinced/thinking/feeling/feeling that others are out to get him/her
Believing/thinking/feeling/feeling that someone is trying to harm him/her
Believing/thinking/feeling/feeling that others want to harm him/her
Believing/thinking/feeling/feeling that others are publicly laughing at him/her
Believing/thinking/feeling/feeling that others are criticizing him/her
Believing/thinking/feeling/feeling that you are under someone else's control
Believing/thinking/feeling/the idea that something happened that has a special meaning to him/her
Talks about Mafia/FBI/UFO
Unusual/strange/bizarre/weird thoughts (contents)

3. Types of delusions
Delusions of grandeur, delusions of persecution, paranoid delusions, delusions of reference, delusions of guilt, delusions of sin, delusions of grandeur, delusions of intoxication, somatic delusions
Is paranoid
Preoccupation with body or mental functioning

2 Hallucinations

Definition

Hallucinations are the perception or experience of things that are not there.

Terminologies and examples

1. Visual hallucinations
Sees animals/black birds/insects at night or evening
Saw people who weren't there
Sees others/people
Had/seen a vision

2. Auditory hallucinations
Hear things that aren't there
Hear voices/sounds
Hear commands
Patient hears voices but cannot indicate what he hears

3. Olfactory hallucinations
Smells strange smells/fire

4. Haptic hallucinations
Feels things that are not there
Feeling something tickling or crawling that isn't there

3 Agitation/aggression

Definition

Agitation entails changes in mood, irritability, and outbursts together with excessive motor activity and restless. Aggression consists of spoken or physical behavior that is threatening or involves harm to someone or something:

Terminologies and examples

1. Physical aggression
Threatening/violent
Grabbing others
Biting, scratching, kicking, hitting, pushing, spiting, attacking, throwing objects, destroying property, slamming doors, breaking things, fighting, fighting, combativity, hurting self/others

2. Verbal aggression
Threaten to hit
Verbally aggressive, swearing, swearing, foul language, insulting, verbal outbursts, screaming, screeching, yelling, shouting, snarling, tantrums, war of words, tantrums, temper tantrums

3. Agitation
Restless/restlessness
Uneasy (depending on context, may also apply to Anxiety or Aberrant motor behavior)
(Psychomotor) agitation
hunted

4. Stubborn/refusal of care
Refuses to wash/dress/take medication
Not cooperating, rebellious, stubborn

5. Hatred
Hate
Contempt

4 Dysphoria/depression

Definition
A depressed mood and/or loss of interest or pleasure.

Terminologies and examples
1. No fun in life
No longer enjoys, hardly enjoys things, enjoys life less, does not find life exciting, no longer experiences pleasure in life, does not enjoy getting up in the morning, anhedonia

2. Suicidality
Suicidal thoughts/plans, think life is not worth living, death wishes, attempted suicide, thoughts about death, suicidal thoughts, internally dissatisfied with my life, dislikes living, convinced that life is no longer worth living, preoccupation with death or suicide, longs for death, would rather be dead, wants to take one's own life

3. Sadness/gloom/depression
Sad, gloomy, depressed mood, sad, often feel down and down, don't feel happy, dejected look, depressed, depressed mood

4. Low self-esteem
Low self-esteem, self-blame, despises self, loses self-confidence, feels like a failure, thinks others see him as a failure, feels inadequate, worth nothing, feels worthless, helplessness and/or misplaced guilt, discouraged

5. Negativity
Recalls bad events/failures/setbacks, pessimistic, sees events from a negative side, worrying about the future, sees the future less bright, no hope for the future, expects the worst for the future, can imagine hardly imagining better times ahead, thoughts of hopelessness, thinks most people are better off than me, often feel hopeless, helpless, worrying, moody, grinding

6. Feeling of emptiness/no feeling
Feeling that his/her life is empty, loss of feeling/feeling nothing anymore

7. Emotional
In tears, tearful, lamenting, sad

9. Social isolation
Social isolation, stopped from many activities and interests, , often bored

10. Delusions consistent with gloom
Preoccupation with depressing subjects: punishment, guilt, death, failure, poverty, nihilistic.

Do not annotate
Vague isolated complaints such as “loneliness”, “burnout”, “boredom”

5 Anxiety

Definition

Emotional response to a perceived or perceived threat that usually leads to avoidance. Often accompanied by physical reactions (tension, trembling, restlessness, shortness of breath) and feelings of nervousness, nervousness, worry or panic.

Terminologies and examples

1. Worry

Worries about physical health, memory, finances, family problems, physical health of relatives, little things, fear of dementia diagnosis, forgetting, the future are annotated, despite the fact that this is sometimes appropriate to the situation.

2. Afraid/Anxiety

Afraid of dementia diagnosis, afraid to fall, don't dare to go out alone, afraid of getting lost, fear of upcoming events, fear of the future, fear of being away from home, fear for health, fear of memory
Generalized anxiety
Fear of being alone/being left alone
He/she finds it scary to…
Anxious, fear, afraid

3. Panic/uncertainty

Trembling
Panic attack
Nervous, nervousness
Hunted, tense, tension
Concerned, Looks worried

4. Phobias

A fear of a certain object or situation and avoiding this object/situation or enduring it with intense fear such as small places of certain animals.

6 Euphoria/elation

Definition

Elevated mood or elation for no apparent reason; optimism or cheerfulness that is out of proportion to the circumstances.

Terminologies and examples

1. Euphoria

Euphoric, euphoria, euphoric feeling
Excessive sense of well-being
Manic
Very cheerful
Excessively energetic
Abnormally good mood
Laugh at something that others don't see the humor in; childish sense of humor; giggle
Roams for multiple days and nights

7 Apathy/indifference

Definition

Apathy is a lack of emotions, motivation or enthusiasm, and a reduced interest in undertaking (social) activities.

Terminologies and examples

1. Withdrawn

He/she is more to himself, he/she is absent more often, more passive, more quiet than usual, indifferent

2. Decreased emotions

Loss or diminished emotion, loss of spontaneous emotion, loss of/diminished emotional responses, unchanging affect, emotional engagement absent, emotional engagement is less, emotionally flattened

Unresponsive / less emotionally

Shows no feeling, less enthusiastic/enthusiasm, flat speech, decline in emotional expressiveness of face, voice and gestures

3. Decreased productivity

Does little, he/she accomplishes little, patient does little during the day, getting fewer things done for the day, less important to complete work or chores, less important for him/her to get things done during the day, does little in a day, less motivation, indecisive, makes no choices/decisions

4. Less participation in social activities/conversations

Interferes less in conversations, participates less in conversations, does not start/less conversations himself/herself, reacts less in a conversation, fewer friends, getting together with friends is less important to him/her, looks less for social contacts/ activities, takes no/less/little part in social activities, less empathetic

5. Loss of initiative

Loss of initiative, lack of initiative/less initiative, less self-initiated behavior, less self-initiation of certain things, someone has to tell her/him what to do every day

6. Loss of interest

No longer interested in things, interested less, less interested in gaining new experiences, less interested in learning new things, spending less time on things that interest him/her, finding less fascinating, putting less effort into something, loss of spontaneous ideas

7. Indifference

Approaches life less intensely, is less concerned about his problems than he should be, when something good happens he becomes less cheerful, laughs/reacts less to positive/negative events

Do not annotate

Vague subtle complaints related to apathy that are mentioned in isolation such as "calmed down", "world has become smaller", and "flat affect"

8 Disinhibition

Definition
Difficulty controlling (verbal, sexual, motor) behavior

Terminologies and examples
1. Inappropriate Comments/Behaviors
More likely to make inappropriate comments to people, inappropriate (sexual) behavior, verbal sexual innuendos, making physical sexual advances, pawing, blunt remarks, saying/doing in public what you normally would not do, painful, talks openly about private matters

2. No consideration for others
Starts talking when others haven't finished speaking, makes hurtful remarks, embarrasses others

3. Risky behavior
Takes more risks than before, reduced risk assessment, behavior that is dangerous to others or oneself, driving recklessly, doing impulsive things

4. Loss of decorum

5. Financially disinhibited
He/she started gambling
Spends a lot of money

9 Irritability/lability

Definition
Easily angered or irritable, very impatient, easily emotionally unbalanced.

Terminologies and examples
1. Irritability
He/she reacts defensively, shows angry/hostile behavior more often, grumpy, more easily irritated, quickly agitated, quickly angry, impatient, gets a little angry if he/she can't come up with a word, easily ends up in an argument with others.

2. Lability
Hot-tempered, bad mood
Quickly emotional, cries more quickly
Quickly Stressed out

10 Aberrant motor behavior

Definition

Excessive repetitive behavior without purpose

Terminology and examples

Not being able to sit still, restless/aimless walking around, wandering, wandering, pacing, repetitive behavior, repeating behavior, repeating questions/sentences, shuffling with feet, picking, wringing hands, fidgeting.

Looking for stuff and rummaging, dressing and undressing

11 Nighttime behavior

Definition

Changes in sleeping behavior during night and the day.

Terminologies and examples

1. Unrested after sleep

Not waking up rested, very tired, sleeping a lot without feeling rested

2. Restless at night

Restless movements in his/her sleep, moves a lot in sleep, scary dreams

3. Sleeping excessively

Sleeps a lot/often, goes to bed much earlier, sleeps in until…, sleeps a lot during the day

4. Difficulty falling/staying asleep

Unable to sleep, difficulty/not falling asleep/falling asleep, deep, difficulty/problems staying asleep, waking up several times/repeatedly during the night

5. Decreased sleep/disturbed sleep

Wakes up earlier, day/night rhythm seems reversed, disturbed day/night rhythm

Do not annotate

Somatic sleep disorders (e.g. apnea) or aids indicative of a sleep disorder (e.g. CPAP, sleep medication)

12 Appetite/eating behavior

Definition
Changes in appetite/weight/eating habits

Terminologies and examples
1. Weight loss/decreased appetite
Got thinner, lost weight, weight loss, appetite reduction/appetite decreased, eats poorly/little, eats less than normal/usually

2. Weight gain/increased appetite
Eats more, gain weight, gain weight

3. Change food preferences
Change of taste, eating more sweet.

Do not annotate
Changes in eating behavior due to somatic disorder or cause

13 General descriptions of neuropsychiatric symptoms

Neuropsychiatric symptoms
Behavioral and psychological symptoms of dementia (BPSD)
Behavioral changes
Changes in character/personality
Challenging behavior
Misunderstood behavior
Problem behavior
Altered behavior
Non-cognitive behaviors
Acts weird
Is not himself/herself anymore
Is another person
Becomes less himself/herself

Do not annotate
Deficits in social cognition
Reduced illness-insight
