# Supplementary material for: The reporting of neuropsychiatric symptoms in electronic health records of individuals with Alzheimer’s disease: a natural language processing study
Source: Alzheimers Res Ther. 2023 May 12;15:94. doi: 10.1186/s13195-023-01240-7 (PMC10176879; doi:10.1186/s13195-023-01240-7)
Supplement: Supplementary file 2 — Additional file 2. Additional analyses. Supplemental Table 1. Number of final annotations, accuracy, and kappa coefficients for the training set and the external test set. Supplemental Table 2. Unadjusted and adjusted prevalence rates of NPS classified in EHRs. Supplemental Table 3. NPS prevalence across EHR based on annotations and classifiers. Supplemental Table 4. NPS classified in EHRs according to sex of the patient. Supplemental Table 5. NPS classified in EHRs according to disease severity. Supplemental Table 6. NPS classified in EHR according to year of visit. Supplemental Table 7. Comparison of NPI assessments between centers. Supplemental Table 8. Kappa coefficients for NPS classified in EHRs vs. NPS reported on NPI according to NPI cut off. [file 13195_2023_1240_MOESM2_ESM.pdf]

Supplemental Table 1. Number of final annotations, accuracy, and kappa coefficients for the training set and the external test set

	Training set (n=500 EHRs)			Test set (n=250 EHRs)		
**NPS category**	**N (%) final annotations**	**Accuracy median [range]**	**Kappa coefficient median [range]**	**N (%) final annotations**	**Accuracy**	**Kappa coefficients**
Hallucinations	18 (3.6%)	0.74 [0.00-0.88]	0.99 [0.98-0.99]	15 (6.0%)	0.98, 0.98	0.39, 0.83
NPS general	45 (9.0%)	0.70 [0.33-0.85]	0.94 [0.92-0.97]	40 (16.0%)	0.90, 0.94	0.64, 0.74
Apathy	122 (24.4%)	0.93 [0.91-0.94]	0.79 [0.70-0.82]	78 (31.2%)	0.84, 0.89	0.62, 0.73
Depression	99 (19.8%)	0.93 [0.86-0.94]	0.76[0.56-0.80]	77 (30.8%)	0.76, 0.81	0.90, 0.92
Sleeping behavior	59 (11.8%)	0.93 [0.90-0.98]	0.63 [0.55-0.86]	45 (18.0)	0.96, 0.96	0.85, 0.86
Disinhibition	44 (8.8%)	0.95 [0.91-0.99]	0.52 [0.44-0.90]	33 (13.2%)	0.91, 0.95	0.57, 0.72
Irritability	89 (17.8%)	0.91 [0.83-0.91]	0.64 [0.41-0.70]	68 (27.2%)	0.88, 0.89	0.61, 0.71
Agitation	38 (7.6%)	0.94 [0.89-0.98]	0.54 [0.38-0.82]	54 (21.6%)	0.90, 0.91	0.71, 0.73
Eating behavior	46 (9.2%)	0.94 [0.88-0.99]	0.64 [0.23-0.85]	34 (13.6%)	0.91, 0.95	0.52, 0.80
Delusions	29 (5.8%)	0.98 [0.97-0.99]	0.79 [0.00-0.91]	16 (6.4%)	0.97, 0.98	0.00, 0.91
Anxiety	122 (24.4%)	0.86 [0.83-0.94]	0.49 [0.00-0.77]	78 (31.2%)	0.88, 0.91	0.66, 0.81
AMB	22 (4.4%)	0.95 [0.93-1.0]	0.35 [0.21-0.65]	18 (7.2%)	0.90, 0.97	-0.02, 0.28
Euphoria	5 (1.0%)	0.99 [0.98-1.0]	0.49 [0.00-0.66]	1 (0.4%)	0.99, 0.99	0.00, 0.80

Abbreviations. AMB = aberrant motor behavior, EHRs = electronic health records, NPS = neuropsychiatric symptoms.

Supplemental Table 2. Unadjusted and adjusted prevalence rates of NPS classified in EHRs

	Alzheimer Center Amsterdam (n=3,001)		Alzheimer Center Erasmus MC (n=646)	
**NPS category**	**Unadjusted prevalence**	**Adjusted prevalence[a]**	**Unadjusted prevalence**	**Adjusted prevalence[a]**
**Apathy**	67.2% [65.5–68.9%]	69.4% [66.9–71.9%]	93.0% [90.9–94.8%]	100.0% [96.1–103.3%]***
**Anxiety**	47.0% [45.2–48.8%]	53.7% [51.0–56.4%]	42.4% [38.6–46.3%]	66.2% [58.8–73.7%]***
**AMB**	63.7% [61.9–65.4%]	47.5% [44.0–50.7%]	81.1% [78.0–84.0%]	267.5% [241.7–291.7%]***[b]
**Irritability**	45.1% [43.3–46.8%]	42.6% [39.8–45.2%]	55.7% [51.9–59.5%]	66.2% [59.8–72.5%]***
**Depression**	29.2% [27.6–30.8%]	38.5% [36.2–40.9%]	63.6% [59.9–67.3%]	75.9% [70.2–81.7%]***
**Disinhibition**	47.1% [45.3–48.9%]	35.4% [32.8–38.1%]	50.5% [46.6–54.3%]	67.6% [60.0–75.1%]***
**Sleeping behavior**	44.8% [43.0–46.6%]	34.5% [31.8–37.2%]	55.6% [51.7–59.4%]	62.2% [55.0–69.3%]***
**Eating behavior**	41.2% [40.3–43.9%]	33.6% [32.2–37.8%]	34.2% [30.6–37.9%]	53.2% [46.4–60.2%]***
**NPS general**	22.1% [20.6–23.6%]	19.6% [17.5–21.7%]	39.0% [35.3–42.8%]	38.7% [33.7–43.7%]***
**Delusions**	24.8% [23.3–26.4%]	17.9% [15.6–20.3%]	51.5% [47.7–55.4%]	62.8% [54.7–71.1%]***[b]
**Agitation**	29.8% [28.2–31.5%]	18.3% [16.0–20.7%]	39.2% [35.5–43.0%]	55.1% [48.1–62.3%]***
**Hallucinations**	10.5% [9.4–11.6%]	8.8% [7.3–10.3%]	16.6% [13.8–19.6%]	35.0% [27.2–43.3%]***[b]

Abbreviations. AMB = aberrant motor behavior, EHR = electronic health record, NPS = neuropsychiatric symptoms.
[a] prevalence estimate corrected for bias due to imperfect test: unadjusted prevalence – (1 – specificity) / (sensitivity + specificity – 1).
[b] Classifier was not included in the analyses as AUC<0.80.
*** FDR-adjusted $p < 0.001$ differences between Alzheimer Center Amsterdam and Alzheimer Center Erasmus MC.

Supplemental Table 3. NPS prevalence across EHR based on annotations and classifiers

NPS category	Training set (n=500 EHRs)			Test set (n=250 EHRs)		
	**Annotations Prevalence**	**Unadjusted classifier prevalence**	**Adjusted classifier prevalence**	**Annotations Prevalence**	**Unadjusted classifier prevalence**	**Adjusted classifier prevalence**
**AMB**	22 (4.4%)	89 (17.8%)	< 0.0%	18 (7.2%)	123 (49.2%)	1.7%
**Agitation**	38 (7.6%)	109 (21.8%)	6.9%	54 (21.6%)	53 (21.2%)	21.1%
**Anxiety**	122 (24.4%)	135 (27.0%)	23.9%	78 (31.2%)	60 (24.0%)	30.8%
**Apathy**	122 (24.4%)	182 (36.4%)	24.1%	78 (31.2%)	138 (55.2%)	30.0%
**Delusions**	29 (5.8%)	80 (16.0%)	4.5%	16 (6.4%)	62 (24.8%)	6.0%
**Depression**	99 (19.8%)	80 (16.0%)	19.1%	77 (30.8%)	87 (34.8%)	30.9%
**Disinhibition**	44 (8.8%)	144 (28.8%)	8.5%	33 (13.2%)	55 (22.0%)	11.8%
**Eating behavior**	46 (9.2%)	123 (24.6%)	8.5%	34 (13.6%)	33 (13.2%)	13.5%
**Hallucinations**	18 (3.6%)	30 (6.0%)	2.7%	15 (6.0%)	12 (4.8%)	2.2%
**Irritability**	89 (17.8%)	143 (28.6%)	17.6%	68 (27.2%)	81 (32.4%)	27.3%
**NPS general**	45 (9.0%)	72 (14.4%)	8.9%	40 (16.0%)	55 (22.0%)	16.0%
**Sleeping behavior**	59 (11.8%)	147 (29.4%)	11.2%	45 (18.0%)	78 (31.2%)	17.0%

Abbreviations. AMB = aberrant motor behavior, EHR = electronic health record, NPS = neuropsychiatric symptoms.
[a] prevalence estimate corrected for bias due to imperfect test: unadjusted prevalence – (1 – specificity) / (sensitivity + specificity – 1).

Supplemental Table 4. NPS classified in EHRs according to sex of the patient

	Alzheimer Center Amsterdam (n=3,001)		Alzheimer Center Erasmus MC (n=646)	
**NPS category**	**Females (n=1,571)**	**Males (n=1,430)**	**Females (n=323)**	**Males (n=323)**
**NPS general**	13.8% [11.8–15.7%]	25.8% [23.6-28.0%]***	33.3% [28.4–38.3%]	44.0% [38.9–49.1%]*
**Agitation**	10.0% [7.7–12.0%]	27.7% [25.3–30.1%]***	43.0% [36.2–49.8%]	67.0% [59.8–74.2%]***
**AMB**	42.5% [39.2–45.8%]	52.9% [49.6–56.2%]***	-	-
**Anxiety**	57.3% [54.6–60.0%]	50.0% [47.2–52.5]***	79.2% [71.7–86.7%]	53.1% [46.0–60.2%]***
**Apathy**	64.3% [61.8–66.8%]	75.3% [72.9–77.6%]***	99.3% [95.4–102.9%]	100.9% [97.4–104.4%]
**Delusions**	18.5% [16.1–20.9%]	17.3% [15.0–19.5%]	-	-
**Depression**	41.3% [38.8–43.8%]	35.4% [33.1–37.8%]**	83.0% [77.3–89.1%]	68.9% [63.0–74.8%]***
**Disinhibition**	32.1% [29.4–34.7%]	39.3% [36.6–41.9%]***	59.6% [52.2–67.1%]	75.5% [68.0–82.9%]***
**Eating behavior**	34.2% [31.5–37.0%]	35.8% [33.0–38.5%]	51.7% [44.9–58.5%]	54.7% [47.7–61.7%]
**Hallucinations**	8.1% [6.6–9.6%]	9.6% [8.0–10.9%]	-	-
**Irritability**	39.5% [36.8–42.2%]	45.8% [43.0–48.5%]**	66.2% [59.8–72.5%]	66.2% [59.8–72.5%]
**Sleeping behavior**	30.8% [28.0–33.5%]	38.8% [36.1–41.5%]***	61.9% [54.8–68.9%]	62.4% [55.4–69.4%]

Abbreviations. AMB = aberrant motor behavior, EHR = electronic health record, NPS = neuropsychiatric symptoms.
Prevalence estimate corrected for bias due to imperfect test: unadjusted prevalence – (1 – specificity) / (sensitivity + specificity – 1).
Classifiers for aberrant motor behavior, delusions, and hallucinations were not used in Alzheimer Center Erasmus MC data as AUC < 0.80.
* FDR-adjusted $p < 0.05$, ** FDR-adjusted $p < 0.01$, *** FDR-adjusted $p < 0.001$.

Supplemental Table 5. NPS classified in EHRs according to disease severity

	**Alzheimer Center Amsterdam (n=3,001)**		**Alzheimer Center Erasmus MC (n=646)**	
**NPS category**	**MCI (n=436)**	**Dementia (n=2,565)**	**MCI (n=157)**	**Dementia (n=489)**
**NPS general**	15.7% [13.8–17.6%]	20.1% [18.1–22.2%]	40.1% [35.1–45.2%]	38.3% [33.2–43.3%]
**Agitation**	19.6% [17.1–22.0%]	18.1% [15.9–20.4%]	53.2% [46.0–60.2%]	55.7% [48.5–62.8%]
**AMB**	50.2% [46.9–53.5%]	46.9% [43.7–50.2%]	-	-
**Anxiety**	61.9% [59.3–64.6%]	52.4% [49.7–55.1%]***	61.7% [54.4–69.0%]	67.5% [60.2–74.8%]
**Apathy**	72.8% [70.4–75.1%]	69.0% [66.5–71.5%]	100.0% [96.3–103.7%]	100.0% [96.3–103.7%]
**Delusions**	7.1% [5.0–9.2%]	21.1% [17.3–22.1%]***	-	-
**Depression**	45.9% [43.4–48.4%]	37.4% [35.0–39.7%]**	89.1% [83.6–94.5%]	71.7% [65.8–77.7%]***
**Disinhibition**	38.7% [36.0–41.3%]	34.9% [32.2–37.5%]	78.6% [71.2–86.3%]	64.1% [56.5–71.8%]**
**Eating behavior**	37.3% [34.5–40.0%]	34.5% [31.8–37.2%]	57.2% [50.2–64.2%]	51.9% [45.3–58.7%]
**Hallucinations**	3.9% [2.7–5.1%]	9.6% [8.1–11.1%]***	-	-
**Irritability**	43.8% [41.1–46.5%]	42.3% [39.5–45.0%]	66.8% [55.5–68.2%]	66.0% [59.7–72.3%]
**Sleeping behavior**	37.3% [34.5–40.0%]	34.1% [31.4–36.8%]	65.4% [58.3–72.4%]	61.1% [54.1–68.1%]

Abbreviations. AMB = aberrant motor behavior, EHR = electronic health record, MCI = mild cognitive impairment, NPS = neuropsychiatric symptoms.
Prevalence estimate corrected for bias due to imperfect test: unadjusted prevalence – (1 – specificity) / (sensitivity + specificity – 1).
Classifiers for aberrant motor behavior, delusions, and hallucinations were not used in Alzheimer Center Erasmus MC data as AUC < 0.80.
* FDR-adjusted $p < 0.05$, ** FDR-adjusted $p < 0.01$, *** FDR-adjusted $p < 0.001$.

Supplemental Table 6. NPS classified in EHR according to year of visit

	**Alzheimer Center Amsterdam (n=3,001)**		**Alzheimer Center Erasmus MC (n=646)**
**NPS category**	**Prevalence 1993-2020 (n=3,001)**	**Prevalence 2004-2020 (n=2,544)**	**Prevalence 2004-2019 (n=646)**
**NPS general**	19.6% [17.5–21.7%]	23.1% [20.7–25.4%][a]	38.7% [33.7–43.7%][d]
**Agitation**	18.3% [16.0–20.7%]	23.3% [20.7–25.9%][c]	55.1% [48.1–62.3%][d]
**AMB**	47.5% [44.0–50.7%]	56.5% [43.7–50.2%][c]	267.5% [241.7–291.7%][d]
**Anxiety**	53.7% [51.0–56.4%]	63.9% [61.0–66.7%][c]	66.2% [58.8–73.7%]
**Apathy**	69.4% [66.9–71.9%]	83.5% [81.2–85.9%][c]	100.0% [96.1–103.3%][d]
**Delusions**	17.9% [15.6–20.3%]	19.1% [16.5–21.7%]	62.8% [54.7–71.1%][d]
**Depression**	38.5% [36.2–40.9%]	43.4% [40.7–46.0%][b]	75.9% [70.2–81.7%][d]
**Disinhibition**	35.4% [32.8–38.1%]	42.9% [40.1–45.7%][c]	67.6% [60.0–75.1%][d]
**Eating behavior**	33.6% [32.2–37.8%]	43.3% [40.5–49.2%][c]	53.2% [46.4–60.2%][d]
**Hallucinations**	8.8% [7.3–10.3%]	10.4% [8.8–12.0%]	35.0% [27.2–43.3%][d]
**Irritability**	42.6% [39.8–45.2%]	52.2% [49.4–55.2%][c]	66.2% [59.8–72.5%][d]
**Sleeping behavior**	34.5% [31.8–37.2%]	41.8% [38.9–44.7%][c]	62.2% [55.0–69.3%][d]

Abbreviations. AMB = aberrant motor behavior, EHR = electronic health record, NPS = neuropsychiatric symptoms.
Prevalence estimate corrected for bias due to imperfect test: unadjusted prevalence – (1 – specificity) / (sensitivity + specificity – 1).
[a] FDR-adjusted $p < 0.05$, [b] FDR-adjusted $p < 0.01$, [c] FDR-adjusted $p < 0.001$, difference between 1993-2020 and 2004-2020 in Alzheimer Center Amsterdam.
[d] FDR-adjusted $p < 0.001$, difference between 2004-2020 in Alzheimer Center Amsterdam and 2004-2019 in Alzheimer Center Erasmus MC.

Supplemental Table 7. Comparison of NPI assessments between centers

NPI domain	Alzheimer Center Amsterdam (n=2,022)	Alzheimer Center Erasmus MC (n=133)
**NPI/NPI-Q,**	0/2,022	9/124
**Any NPS**	1625 (80.4%)	112 (84.2%)
**AMB**	348 (17.2%)	29 (22.0%)
**Agitation**	326 (16.1%)	36 (27.2%)***
**Anxiety**	603 (29.8%)	52 (39.1%)*
**Apathy**	1081 (53.5%)	61 (46.2%)
**Delusions**	157 (7.8%)	32 (24.1%)***
**Depression**	646 (31.9%)	74 (55.6%)***
**Disinhibition**	259 (12.8%)	34 (25.8%)***
**Eating behavior**	580 (28.7%)	52 (39.1%)*
**Hallucinations**	90 (1.2%)	15 (11.3%)***
**Irritability**	783 (38.7%)	63 (47.7%)
**Sleeping behavior**	407 (20.1%)	41 (31.8%)**

Abbreviations. AMB = aberrant motor behavior, NPI = Neuropsychiatric Inventory, NPI-Q = Neuropsychiatric Inventory questionnaire, NPS = neuropsychiatric symptoms.
Data presented as N (%).
* FDR-adjusted $p < 0.05$, ** FDR-adjusted $p < 0.01$, *** FDR-adjusted $p < 0.001$.

Supplemental Table 8. Kappa coefficients for NPS classified in EHRs vs. NPS reported on NPI according to NPI cut off

	**Alzheimer Center Amsterdam**		**Alzheimer Center Erasmus MC**	
**NPS category**	**NPI≥1**	**NPI≥4**	**NPI≥1**	**NPI≥4/NPI-Q≥2**
**Agitation**	κ=0.18	κ=0.09	κ=0.26	κ=0.13
**AMB**	κ=0.03	κ=0.02	-	-
**Anxiety**	κ=0.15	κ=0.09	κ=0.01	κ=0.06
**Apathy**	κ=-0.02	κ=-0.02	κ=-0.03	κ=-0.03
**Delusions**	κ=0.12	κ=0.10	-	-
**Depression**	κ=0.28	κ=0.20	κ=-0.04	κ=0.02
**Disinhibition**	κ=0.07	κ=0.04	κ=0.09	κ=0.07
**Eating behavior**	κ=0.12	κ=0.09	κ=0.14	κ=0.13
**Hallucinations**	κ=0.18	κ=0.11	-	-
**Irritability**	κ=0.11	κ=0.08	κ=0.11	κ=0.12
**Sleeping behavior**	κ=0.05	κ=0.04	κ=0.13	κ=0.09

Abbreviations. AMB = aberrant motor behavior, EHR = electronic health record, NPS = neuropsychiatric symptoms.
Classifiers for aberrant motor behavior, delusions, and hallucinations were not used in Alzheimer Center Erasmus MC data as AUC < 0.80.
